# Effect of Baseline HIV Disease Parameters on CD4+ T Cell Recovery After Antiretroviral Therapy Initiation in Kenyan Women

**DOI:** 10.1371/journal.pone.0011434

**Published:** 2010-07-02

**Authors:** Lyle R. McKinnon, Makobu Kimani, Charles Wachihi, Nico J. Nagelkerke, Festus K. Muriuki, Anthony Kariri, Richard T. Lester, Lawrence Gelmon, T. Blake Ball, Francis A. Plummer, Rupert Kaul, Joshua Kimani

**Affiliations:** 1 Department of Medicine, University of Toronto, Toronto, Canada; 2 Department of Medical Microbiology, University of Nairobi, Nairobi, Kenya; 3 Department of Community Medicine, United Arab Emirates University, Al Ain, United Arab Emirates; 4 Department of Medical Microbiology, University of Manitoba, Winnipeg, Canada; 5 Department of Immunology, University of Manitoba, Winnipeg, Canada; 6 Laboratory of HIV Immunology, Public Health Agency of Canada, Winnipeg, Canada; 7 National Microbiology Lab, Public Health Agency of Canada, Winnipeg, Canada; University of California San Francisco, United States of America

## Abstract

**Background:**

Antiretroviral therapy (ART) for HIV infection reconstitutes the immune system and improves survival. However, the rate and extent of CD4+ T cell recovery varies widely. We assessed the impact of several factors on immune reconstitution in a large Kenyan cohort.

**Methodology/Principal Findings:**

HIV-infected female sex workers from a longitudinal cohort, with at least 1 year of pre-ART and 6 months of post-ART follow-up (n = 79), were enrolled in the current study. The median pre-ART follow-up was 4,040 days. CD4 counts were measured biannually and viral loads where available. The median CD4 count at ART initiation was 180 cells/ul, which increased to 339 cells/ul at the most recent study visit. The rate of CD4+ T cell increase on ART was 7.91 cells/month (mean = 13, range −25.92 to 169.4). LTNP status prior to ART initiation did not associate with the rate of CD4 recovery on ART. In univariate analyses, associations were observed for CD4 recovery rate and duration of pre-ART immunosuppression (r = −0.326, p = 0.004) and CD4 nadir (r = 0.284, p = 0.012). In multivariate analysis including age, CD4 nadir, duration of HIV infection, duration of pre-ART immunosuppression, and baseline viral load, only CD4 nadir (p = 0.007) and not duration of immunosupression (p = 0.87) remained significantly associated with the rate of CD4 recovery.

**Conclusions/Significance:**

These data suggest that prior duration of immune suppression does not predict subsequent recovery once ART is initiated and confirm the previous observation that the degree of CD4 depletion prior to ART initiation is the most important determinant of subsequent immune reconstitution.

## Introduction

Since access to ART to treat HIV-1 infection has increased, life expectancy for people living with HIV has increased dramatically, from 5–10 years in the absence of treatment, to more than 25 years following HIV infection if treated appropriately [1]. ART decreases the risk of opportunistic infections following suppression of HIV replication, in conjunction with reconstitution of blood CD4+ T cell counts, partially reversing the hallmark immunodeficiency that develops during AIDS [2], [3]. This immune recovery means that co-infection prophylaxis can be safely discontinued in most successfully treated individuals [4].

However, not all individuals on ART reconstitute CD4+ T cells at the same rate, or to the same extent. As such, as many as 30% of patients who start ART are classified as immunological non-responders [5]. Factors that predict of poor immune reconstitution include a lower CD4 nadir [6], [7], older age [8], increased immune activation [9], [10], altered T cell homeostasis [7], [11], [12], markers of microbial translocation [13], and HIV co-receptor usage [14]. One US-based study demonstrated that individuals with a lower baseline CD4 count at the time of ART initiation achieved less immune reconstitution than those in the higher CD4 strata, and this was associated with reduced naïve∶memory CD4 cell and CD4∶CD8 cell ratios [15]. To date, of many factors associated with ART recovery, CD4 nadir has been shown to be the most consistent predictor of subsequent immune reconstitution. As a result, absolute blood CD4+ T cell count is the key determinant in the timing of ART initiation, with current World Health Organization guidelines suggesting that treatment should start before CD4≤350/ul.

Rates of CD4+ decline are variable, and in the absence of ART some individuals remain relatively well for long periods despite low CD4+ T cell counts. In addition, some subjects can survive for several years with low CD4 counts. We hypothesized that prior HIV disease progression status and/or duration of pre-ART immunosuppression (defined as the length of time with CD4≤200 prior to ART initiation) would constitute additional independent predictors of poor immune reconstitution on ART, in addition to the nadir CD4+ T cell count, and might need to be considered by ART providers.

## Methods

We examined this question in a longitudinal female sex worker cohort where an ART program was initiated in 2005 as a part of the U.S. President's Emergency Plan for AIDS Relief (PEPFAR). All subjects gave informed written consent prior to participation in the study, and Institutional Review Boards at Universities of Manitoba and Toronto, and Kenyatta National Hospital (Nairobi, Kenya), approved the study. Testing of viral loads was not standard of care in this clinic during the time that the study was conducted, though CD4 counts were typically measured twice annually. All patients in the study received the standard first-line therapy of Triomin (3TC, D4T, NVP). At the time of analysis, 177 subjects were receiving ART through this program. Because the aim was to determine factors influencing the rate of CD4+ T cell recovery on ART, we included only those participants with at least 365 days of pre-ART follow-up and 180 days of post-ART follow-up (n = 79). Participants were called long-term non-progressors (LTNP) if they had been HIV+ for >10 years with CD4 counts >500 cells/ul. Median HIV-positive follow-up prior to ART initiation was 4,448 days (mean 4,040, range 462–7,922 days), and median follow-up post-ART initiation was 697 days (mean 758, range 187–2,105 days). Univariate correlations were preformed using Spearman's Rank Correlation. Multivariate analyses included Linear Regression with CD4 recovery/month and CD4 recovery as independent variables, and controlling for a number of variables predicted to have an effect of the rate of CD4 recovery following ART initiation (described below).

## Results

Most subjects showed evidence of a CD4+ T cell increase after ART initiation; the median CD4 count at ART initiation was 180 cells/ul (mean 211, range 3–851 cells/ul), and this increased to 339 cells/month at the most recent available CD4 count. The total duration of ART was correlated with total CD4 count recovery (r = 0.48, p<0.0001, Spearman rank correlation). The rate of CD4+ T cell increase on ART was 7.91 cells/month (mean = 13, range −25.92 to 169.4). This rate slowed significantly over time, as demonstrated by the inverse relationship between recovery/month and length of time on ART (r = −0.36, *P*<0.0001). This reflects the more rapid recovery of CD4 cells early in ART, as has been previously described [5].

**Figure 1:**
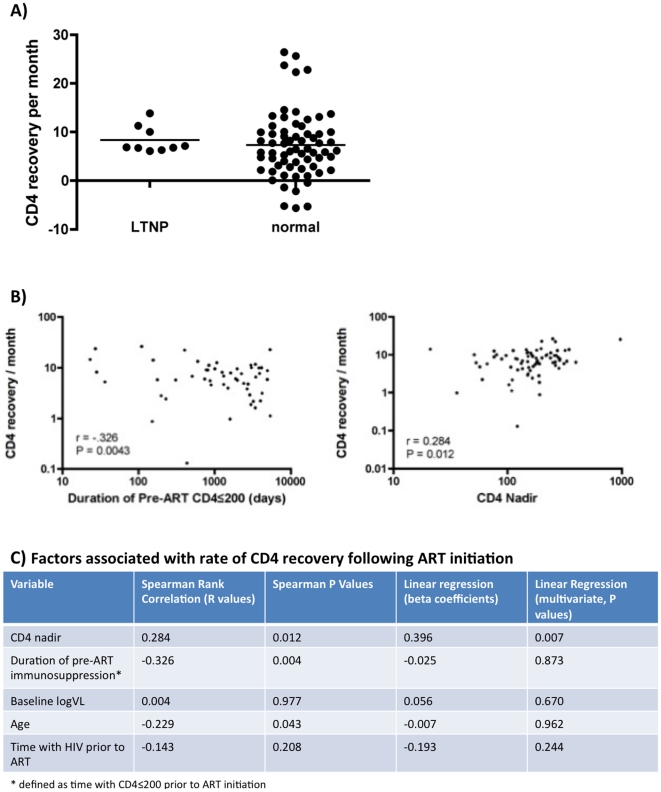
Correlations of pre-ART HIV disease progression and subsequent immune reconstitution following ART initiation in a Kenyan female sex worker cohort. A) Prior disease progression status does not correlate with the rate of CD4 recovery. Long-term non-progressors (LTNPs) are compared to normal progressors (those who did not meet the definition of LTNP). B) In univariate analyses, re-ART duration of immunosuppression (CD4<200) was inversely associated with CD4 recovery/month (r = −0.326, p = 0.004), while CD4 nadir correlated with CD4 recovery/month (r = 0.284, p = 0.012). On x-axis is either pre-ART duration of immunosupression (days) or CD4 nadir, while rate of CD4 recovery is on the y-axis (increase in cells/month). C) CD4 nadir (p = 0.007), but not length of immunosuppression (p = 0.873), was associated with the rate of CD4 recovery in multivariate analysis.

We investigated the association of several factors with the rate of CD4 recovery, including age, CD4 nadir (defined as second lowest CD4 count), baseline VL (available for 60/79 participants), duration of pre-ART immunosuppression, duration of time with HIV infection, and prior status as a long-term non-progressor (HIV+ for ≥10 years with CD4 ≥500). Of subjects with adequate pre- and post-ART follow-up, we identified several “prior” LTNPs (n = 10). Although LTNPs had a trend towards a higher CD4 Nadir (median 239 vs. 160 cells/ul, p = 0.137), no differences in the rate of CD4 recovery following ART were observed (Figure 1A, p = 0.25, Mann Whitney U test). We then calculated the time from the first CD4 count ≤200/ul to ART initiation, and correlated this to the subsequent rate of immune reconstitution. Since an ART program was initiated in this cohort 20 years after its inception (1985–2005), immunosuppression prior to ART initiation was common in this population, ranging from 0 to 5,357 days from first CD4<200 to ART initiation (mean 1,568 days, median 896 days). While no correlation was observed between duration of pre-ART immunosuppression and total CD4 recovery, immunosuppression was inversely correlated to the rate of CD4 recovery (Figure 1B, r = −0.326, p = 0.004). In univariate analyses, subjects with longer duration of pre-ART immunosuppression were also older (r = 0.326, p = 0.004) and had a lower CD4 nadir (r = −0.652, p<0.001). As expected, there was also an association between CD4 nadir and the rate of CD4 recovery (Figure 1B, r = 0.284, p = 0.012).

Because many factors are known to influence the CD4 reconstitution on ART, we used linear regression to determine which variables were independently associated with the rate of CD4 recovery. In a model that included CD4 nadir, age, baseline viral load, time with HIV infection, and time with low CD4 counts prior to ART initiation, only CD4 nadir remained significantly associated with a lower rate of CD4 recovery (Figure 1C, p = 0.007). Duration pre-ART immunosuppression was no longer associated with the subsequent rate of CD4 recovery in this model (p = 0.87). To determine whether the overall extent of CD4 recovery might be predicted by duration of pre-ART immunosuppression, we also ran similar models using final CD4 count was used as the main outcome of ART recovery, and obtained similar results (not shown).

Because CD4 counts increase more dramatically in the early stages of ART, we also analyzed our data by CD4 gains made over the first year (“early” recovery) and those made during the remainder of follow-up (late recovery). Although the previous associations were not observed with early recovery, linear regression analyses also found that only CD4 nadir was associated with late CD4 recovery rate (p = 5×10^−5^, data not shown).

## Discussion

ART increases the length and quality of life of HIV-infected subjects by reversing the immunodeficiency characteristic of AIDS, but there is heterogeneity in the rate and extent to which immune recovery is realized. In the present study, we evaluated whether prior disease progression status (LTNP) or duration of pre-ART immunosuppression predicted an altered rate of recovery following ART initiation in a cohort where ART was scarcely available from 1985–2004. We based this hypothesis on the rationale that LTNPs and/or patients with limited amounts of time with low CD4 counts would better retain key immunological features, such as lymphoid architecture and T cell homeostasis, and this in turn would allow their CD4+ T cell counts to recover more rapidly. A second possibility is that whatever factors caused these subjects to be LTNPs in the first place may lead to a more rapid CD4 constitution once viral load was suppressed by ART. In contrast, a recent study of HIV controllers placed on ART showed that these individuals reconstituted CD4+ T cells slower than average, possibly due the their longer time of infection prior to ART initiation [16]. However, in our study, while the duration of pre-ART immunosuppression was strongly associated with slower subsequent CD4 recovery in univariate analysis, the only independent predictor of slow recovery in multivariate models was CD4 nadir. Therefore, these data confirm the previous observation that the degree of CD4 depletion prior to ART initiation is the most consistent determinant of subsequent immune reconstitution, representing one of the first studies to confirm this in sub-Saharan Africa. These data also suggest that once a patient progresses to a certain level of CD4 depletion, it may not be as critical for how long they have been at that level, perhaps because at this point, substantial immunological damage has already occurred.

Given recent studies showing that starting ART at higher CD4 counts (≥350–500/ul) is both clinically advantageous and leads to improved immune constitution [17], [18], the current data add to the growing body of knowledge that delaying ART may severely limit the clinical impact of therapy. Because delayed or lack of an immunological response to ART is multi-factorial [5], further investigations are needed to understand what influences better response to ART, to early on identify patients whose benefit from starting ART might be diminished, and to develop alternative therapeutic strategies. Furthermore, given the late presentation of many HIV-infected individuals to ART programs in the developing world, these data emphasize the need to further promote HIV testing and counseling in order to facilitate earlier ART initiation, not only to potentially prevent further transmission [19], but also to maximize clinical benefit to the patient.
